# Cost-utility analysis of sutureless and rapid deployment versus conventional aortic valve replacement in patients with moderate to severe aortic stenosis in Thailand

**DOI:** 10.1371/journal.pone.0296875

**Published:** 2024-01-19

**Authors:** Unchalee Permsuwan, Seri Singhatanadgige, Kawinnooch Boonpipattanapong, Worawong Slisatkorn, Angsu Chartrungsan, Prompak Nitayavardhana, Nutthawadee Luangthong, Pramote Porapakkham, Jirawit Yadee

**Affiliations:** 1 Center for Medical and Health Technology Assessment (CM-HTA), Department of Pharmaceutical Care, Faculty of Pharmacy, Chiang Mai University, Chiang Mai, Thailand; 2 Department of Pharmaceutical Care, Faculty of Pharmacy, Chiang Mai University, Chiang Mai, Thailand; 3 Division of Cardiothoracic Surgery, Department of Surgery, Faculty of Medicine, Chulalongkorn University, Bangkok, Thailand; 4 Division of Cardio-Thoracic Surgery, Department of Surgery, Faculty of Medicine Siriraj Hospital, Mahidol University, Bangkok, Thailand; 5 Department of Cardiothoracic Surgery, Central Chest Institute of Thailand, Nonthaburi, Thailand; 6 Ph.D. Degree Program in Pharmacy, Faculty of Pharmacy, Chiang Mai University, Chiang Mai, Thailand; BSMMU: Bangabandhu Sheikh Mujib Medical University, BANGLADESH

## Abstract

**Background:**

Sutureless and rapid deployment aortic valve replacement (SUAVR) has become an alternative to conventional aortic valve replacement (CAVR) for aortic stenosis (AS) treatment due to its advantages in reducing surgery time and improving outcomes. This study aimed to assess the cost-utility of SUAVR vs. CAVR treatment for patients with moderate to severe AS in Thailand.

**Methods:**

A two-part constructed model was used to estimate the lifetime costs and quality-adjusted life years (QALYs) from both societal and healthcare perspectives. Data on short-term mortality, complications, cost, and utility data were obtained from the Thai population. Long-term clinical data were derived from clinical studies. Costs and QALYs were discounted annually at 3% and presented as 2022 values. The incremental cost-effectiveness ratio (ICER) was calculated to determine additional cost per QALY gained. Deterministic and probabilistic sensitivity analyses were performed.

**Results:**

SUAVR treatment incurred higher costs compared with CAVR treatment from both societal (THB 1,733,355 [USD 147,897] vs THB 1,220,643 [USD 104,150]) and healthcare provider perspectives (THB 1,594,174 [USD 136,022] vs THB 1,065,460 [USD 90,910]). In addition, SUAVR treatment resulted in lower health outcomes, with 6.20 life-years (LYs) and 4.95 QALYs, while CAVR treatment achieved 6.29 LYs and 5.08 QALYs. SUAVR treatment was considered as a dominated treatment strategy using both perspectives. Sensitivity analyses indicated the significant impact of changes in utilities and long-term mortality on the model.

**Conclusion:**

SUAVR treatment is not a cost-effective treatment strategy compared with CAVR treatment for patients with moderate-severe AS in Thailand, as it leads to higher costs and inferior health outcomes. Other important issues related to specific patients such as those with minimally invasive surgery, those undergoing AVR with concomitant procedures, and those with calcified and small aortic root should be taken into account.

## Introduction

Aortic stenosis (AS) is a public health concern that is expected to increase with population aging. Among elderly patients, the prevalence of AS is increasing, with 12.4% of patients over 75 years of age [1]. If left untreated, severe AS carries a poor prognosis, with a mortality rate of 30–50% [2]. The standard approach for treating patients with severe or symptomatic AS is aortic valve replacement (AVR) [3, 4]. However, sutureless and rapid-deployment AVR (SUAVR) has emerged as an alternative to conventional AVR (CAVR) to treat AS. The aim of the SUAVR device is to reduce surgery time, enhance valve insertion, and improve surgical outcomes. Furthermore, this device also facilitates the removal of the diseased valve, decalcification of the annulus, and direct visualization during the implantation process [5, 6].

Currently, two categories are available regarding SUAVR devices, specifically LivaNova’s Perceval S and Edwards Intuity System. The Perceval S System is a self-expanding, stentless, and sutureless valve, while the Edwards Intuity System is a balloon-expandable, stented valve. Perceval S relies on its inherent design for deployment, whereas Edwards Intuity uses a balloon for expansion and valve securing. One related efficacy study indicated a similar rate of 30-day mortality between SUAVR and CAVR [7]. Comparable mortality rates between the Perceval S and Intuity Elite System were also reported [7, 8]. In addition, SUAVR had significantly shorter cardiopulmonary bypass (CPB) and aortic cross clamp (ACC) times compared with CAVR [7]. Several studies have examined the economic impact of SUAVR treatment. The findings across three studies revealed that SUAVR treatment is cost-saving compared with CAVR treatment based on the cost-effectiveness results [9–11].

In Thailand, the limited availability of healthcare resources necessitates generating country-specific evidence to justify the cost-effectiveness of costly health technologies including drugs, vaccines, and medical devices. This economic evidence plays an important role in supporting decision-making. To evaluate the cost-effectiveness of these costly healthcare technologies, various stakeholders, including the Subcommittee for the Development of the Benefit Package and Service Delivery (SCBP), require health economic evaluations like cost-utility and cost-effectiveness analyses. When these costly healthcare technologies are approved for including into the Universal Health Coverage Benefit Package (UHCBP) under the Universal Health Coverage Scheme (UHCS), patients will not be required to pay for this benefit package. SUAVR treatment has been purposed and systematically prioritized based on predetermined criteria by a selection working group under the SCBP. To provide valuable insights that support evidence-based decision-making and rationally optimize healthcare resource allocation, this study aimed to compare the cost-utility of SUAVR vs. CAVR treatment for patients with moderate to severe AS in Thailand.

## Materials and methods

### Model description

**Fig 1:**
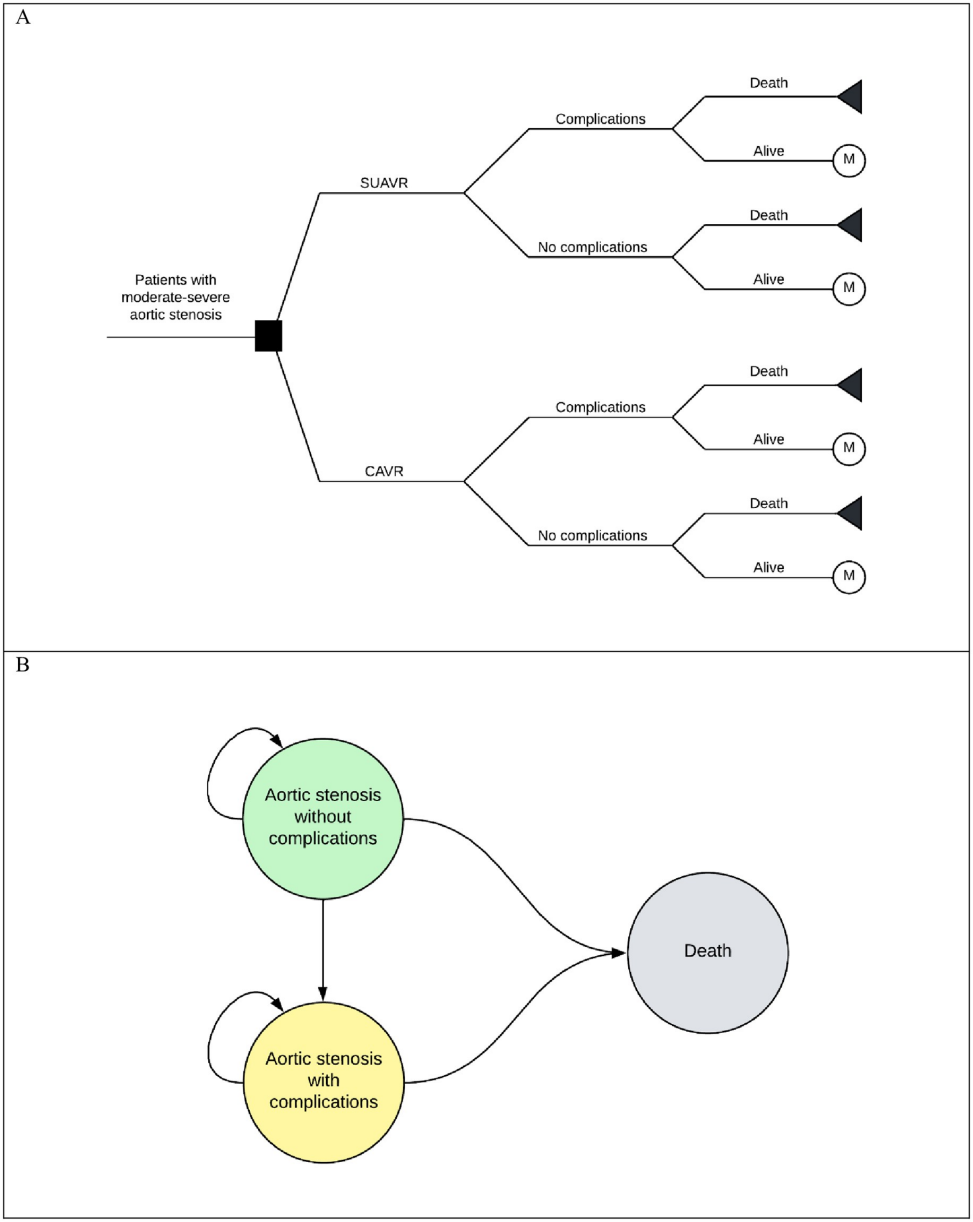
The two-part constructed model. Abbreviations: CAVR, conventional bioprosthetic aortic valve replacement; SUAVR, sutureless/rapid-deployment aortic valve replacement.

The study used a two-part constructed model including a decision tree and a Markov model, which was integrated with Microsoft Excel for Microsoft 365 (Microsoft Corporation, Redmond, WA, USA) (Fig 1). This model was designed to assess both short-term (30-day) and long-term outcomes following the intervention. The study population consisted of individuals undergoing either SUAVR or CAVR treatment. Within the initial 30-day period, patients were categorized as either alive or deceased after receiving the intervention (Fig 1A). Among those who survived, some patients were discharged without any complications, while other patients experienced early complications, including stroke, atrial fibrillation, acute kidney injury, major bleeding, permanent pacemaker implantation, and paravalvular leakage. Following the initial short-term decision tree model, patients would transition to the long-term Markov model (Fig 1B). This model included three health states: AS without complication, AS with complications, and death. Patients not experiencing any complications would enter the "AS without complication" health state in the Markov model. On the other hand, patients encountering early complications would transition to the "AS with complications" health state within the Markov model. The Markov model performed with a cycle length of one year and a lifetime horizon, considering that all patients would eventually reach the absorbing health state of death.

### Intervention and comparator

The intervention in this study was SUAVR treatment with either LivaNova’s Perceval S or Edwards Intuity Valve. The comparator in this study comprised CAVR treatment.

### Population

The study population comprised patients with moderate to severe AS. The starting patient age was 65 years reflecting the general practice in Thailand.

### Data collection

To obtain the direct medical costs, direct non-medical costs and utilities, the data were collected from patients receiving a diagnosis of moderate to severe AS and undergoing either SUAVR or CAVR treatment between January 2015 and June 2023. The data were sourced from three large hospitals, including two university-affiliated hospitals located in Bangkok, and a government-affiliated specialized hospital for cardiovascular and pulmonary care located in Nonthaburi, near Bangkok. To minimize potential selection bias, propensity score matching was performed to identify matched pairs of patients undergoing either SUAVR or CAVR treatment using various risk factors. A propensity score was generated for each patient using a nonparsimonious multivariable logistic regression model considering factors including age, sex, severity of AS, surgical risk assessment based on the European System for Cardiac Operative Risk Evaluation II (EuroSCORE II), left ventricular ejection fraction, AVR with isolated or concomitant procedure, and the New York Heart Association (NYHA) functional class. A greedy nearest-neighbor matching approach without replacement, with a 1:1 matching ratio was performed to ensure that each patient with SUAVR was paired with a patient with CAVR having a similar propensity score. The analysis was performed using STATA Software, Version 14.0 (StataCorp. 2015. Stata Statistical Software: Release 14. College Station, TX: StataCorp LP). Consequently, 60 matched cohorts of patients with AS who underwent either SAVR or TAVR treatments were identified.

The data regarding direct medical costs were extracted from the electronic databases of the three hospitals, where information was gathered from matched cohorts of patients with AS who underwent either SUAVR or CAVR treatments. As for the direct non-medical costs and utilities, these were obtained through interviews with patients who underwent SUAVR or CAVR treatment or were under follow-up care at the three hospitals during the recruitment period, which extended over approximately six months from January to June 2023.

### Transitional probabilities

#### Short-term clinical outcomes

The short-term clinical outcomes included death and in-hospital complications during 30 days after surgery. The in-hospital complications included stroke, acute kidney injury, atrial fibrillation, major bleeding, permanent pacemaker implantation, and paravalvular leakage. Transitional probabilities of death and complications for the CAVR group were derived from three hospital databases. Patients were also classified into three groups: 1) patients with overall AVR, 2) patients with isolated AVR (AVR without concomitant procedure), and 3) patients with combined AVR (AVR with concomitant procedures).

To obtain the risk of death and in-hospital complications of the SUAVR group, systematic review and meta-analysis were conducted. In brief, a systematic literature search was conducted in four databases (PubMed, Scopus, Web of Science, and Embase) covering articles from inception to December 2022. The primary search terms were “sutureless”, “rapid deployment”, “aortic valve replacement”, and “aortic stenosis”. Detailed information regarding the search strategies can be found in S1 Table in S1 File. To be eligible for inclusion in this review, the article had to meet the following inclusion criteria: 1) randomized controlled trial (RCT) or propensity-score matched cohort study of SUAVR vs. CAVR treatment; 2) the study participants comprised patients with moderate and/or severe AS, and 3) clinical outcomes were reported at 30 days. Ahead-of-print articles were also considered. The review excluded other study types, such as non-randomized controlled trial, observational study without propensity-score matching, case report, and review article. The flow diagram for systematic literature review is provided in S1 Fig in S1 File. Quality assessment for randomized controlled trials was performed using the Revised Cochrane risk of bias tool for randomized trials (RoB) version 2.0 [12]. In addition, the Risk Of Bias In Non-randomized Studies—of Exposure (ROBINS-E) tool [13] was used to assess the risk of bias for propensity-score matched cohort study. The results of quality assessment are shown in S3 and S4 Tables in S1 File.

In total, 4 RCTs [14–17] and 22 propensity-score matched cohort studies [7, 18–38] were identified, as detailed in S2 Table in S1 File. Then, the meta-analysis using the random-effects model was performed to estimate the relative risk (RR) of the interested outcomes for patients with AS undergoing SUAVR compared with CAVR treatment. This approach was selected by the inherent differences in study design, which could contribute to heterogeneity in the results. The details on RR estimation are shown in S5 Table in S1 File. Those RRs were applied with the mortality risk and in-hospital complications of the CAVR group to generate the transitional probabilities of death and in-hospital complications of the SUAVR group. Clinical inputs at 30 days following SUAVR and CAVR treatments are listed in Table 1.

**Table 1:** Clinical input parameters.

Parameters	Value (standard error)		Source(s)
	SUAVR	CAVR	
***30-day outcomes*** * **: *Overall AVR***			
Complication	0.2901 (0.2753–0.3049)	0.2795 (0.2652–0.2937)	CAVR: Data collection from three hospital databases (as stated).^#^ SUAVR: Calculation using relative risk^$^ from a meta-analysis of included clinical studies [7, 14–38].
Death in no-complication group	0.0294 (0.0279–0.0309)	0.0303 (0.0288–0.0318)	
Death in complication group	0.1252 (0.1188–0.1315)	0.1290 (0.1224–0.1356)	
***30-day outcomes*** * **: *Isolated AVR***			
Complication	0.2769 (0.2628–0.2910)	0.2636 (0.2502–0.2771)	
Death in no-complication group	0.0117 (0.0111–0.0122)	0.0125 (0.0119–0.0131)	
Death in complication group	0.0000	0.0000	
***30-day outcomes*** * **: *Combined AVR***			
Complication	0.3096 (0.2938–0.3254)	0.2975 (0.2823–0.3127)	
Death in no-complication group	0.0462 (0.0439–0.0486)	0.0471 (0.0447–0.0495)	
Death in complication group	0.2311 (0.2193–0.2428)	0.2353 (0.2233–0.2473)	
***1-year outcomes***			
Mortality	0.0384 (0.0364–0.0404)	0.0343 (0.0326–0.0361)	Borger MA [15], Fischlein T [17], Ono Y [36]
Stroke	0.0214 (0.0203–0.0225)	0.0197 (0.0187–0.0207)	
Atrial fibrillation	0.0393 (0.0373–0.0413)	0.0922 (0.0875–0.0969)	
Major bleeding	0.0327 (0.0310–0.0344)	0.0338 (0.0321–0.0355)	
Acute kidney injury	0.0218 (0.0207–0.0229)	0.0207 (0.0196–0.0218)	
Pacemaker implantation	0.0405 (0.0384–0.0426)	0.0289 (0.0274–0.0304)	
Paravalvular leakage	0.0858 (0.0814–0.0902)	0.0289 (0.0274–0.0304)	

Abbreviations: AVR, aortic valve replacement; CAVR, conventional bioprosthetic aortic valve replacement; RR, relative risk; SUAVR, sutureless and rapid deployment aortic valve replacement

* The complications included stroke, acute kidney injury, atrial fibrillation, major bleeding, permanent pacemaker implantation, and paravalvular leakage.

^#^ The transitional probabilities were derived from three hospital databases.

^$^ Relative risks from a meta-analysis of included studies were applied with the mortality risk and in-hospital complications of the CAVR group to generate the transitional probabilities of the SUAVR group.

#### Long-term clinical outcomes

Long-term clinical outcomes were also obtained through a systematic review and meta-analysis of RCT and propensity-score matched cohort studies comparing SUAVR vs. CAVR treatment. The inclusion criteria considered articles reporting clinical outcomes at one year or beyond. Two RCTs [15, 17] and a propensity-score matched cohort study [36] were included in the analysis for 1-year clinical outcomes (S1 Fig in S1 File). Characteristics of included studies are provided in S2 Table in S1 File. Pooled analyses for combining outcomes of all-cause death and complications at 1 year after SUAVR or CAVR treatment were performed to generate the transitional probability data in the model. These 1-year probabilities were subsequently carried forwarded throughout the model’s time horizon. Beyond the initial 1-year period, the model applied the age-specific mortality rates (ASMR) for the Thai general population, using data from the Global Health Observatory of the World Health Organization [39]. Long-term clinical inputs are listed in Table 1.

#### Costs

The costs included in this study were direct medical and direct non-medical costs. However, according to the Thai health technology assessment (HTA) guideline, indirect costs were not considered to avoid double counting of benefits in terms of costs and effectiveness of the health intervention [40]. Both societal and healthcare system perspectives were considered in the analyses. The direct medical costs included costs of aortic valve, materials, anesthetic, drugs, laboratory tests, intensive care unit stays, hospital stays, blood products, imaging, special diagnostic procedures, and instruments. These cost data were derived from the hospitals’ electronic databases, which collected data from matched cohorts of patients with AS undergoing SUAVR or CAVR treatments in the three hospitals.

For the costs related to complications and follow-up treatments, the study obtained the data from the electronic claim (e-Claim) database, which is managed by the National Health Security Office, Thailand. This database contains data of all Thai patients receiving healthcare services under the UHCS, providing coverage for approximately 70% of the entire population. The study included hospital records from patients aged ≥18 years and admitted with a primary diagnosis of AS from 2015 to 2022. The diagnoses recorded in the e-Claim system followed the World Health Organization’s International Classification of Diseases, 10th Revision (ICD-10). Specifically, a diagnosis of AS was represented by the code I350. The ICD-10 codes were also used to identify complications, including stroke (I60X, I61X, I62X, I63X), atrial fibrillation (I480, I481, I482, I489), major bleeding (K920, K922, J942, K661), acute renal failure (N17X), pacemaker implantation (Z950), and paravalvular leakage (T820, T829). By using these ICD-10 codes, the study was able to assess the costs associated with complications and follow-up treatments. In order to ensure patient confidentiality, distinct encoded identifiers were used to link data from the e-Claim database, encompassing data regarding hospital admissions, mortality, and costs associated with hospitalization.

In terms of direct non-medical costs, costs of accommodation, food, transportation, and caregivers were directly collected by interviewing the patients who underwent SUAVR or CAVR treatment at the three hospitals.

All cost data were adjusted for inflation using the medical care section of Thailand’s consumer price index [41] and presented in the year 2022 in Table 2. Additionally, the cost data were converted from Thai Baht (THB) into the United States dollars (USD) using a 2022 Purchasing Power Parity (PPP) conversion factor of 11.72 for Thailand and 1.00 for the United States (US), as provided by the International Bank for Reconstruction and Development [42].

**Table 2:** Costs and utility inputs.

Parameters	Value (standard error)		Data distribution	Source(s)
	SUAVR	CAVR		
***Direct medical costs***				
Costs of surgery admission (THB)				
Valve	458,818 (367,055–550,582)	137,186 (109,749–164,623)	Gamma	Hospital database
Valve-related materials	36,889 (29,511–44,267)	41,526 (33,221–49,832)	Gamma	
Anesthesia and operation	146,881 (117,504–176,257)	149,003 (119,202–178,803)	Gamma	
Drugs	73,172 (53,331–93,013)	76,813 (61,519–92,108)	Gamma	
Laboratory tests	66,276 (55,129–77,422)	69,713 (60,600–78,826)	Gamma	
Hospital stays	19,961 (15,969–23,953)	20,500 (16,400–24,600)	Gamma	
Imaging procedures	20,342 (16,135–24,549)	15,815 (12,361–19,270)	Gamma	
Special diagnosis procedures	13,080 (11,686–14,473)	10,486 (8,999–11,973)	Gamma	
Medical instruments	77,406 (66,823–87,989)	77,500 (67,298–87,702)	Gamma	
Blood products	43,429 (35,728–51,129)	51,637 (43,990–59,284)	Gamma	
Rehabilitation	2,449 (1,929–2,969)	2,060 (1,600–2,520)	Gamma	
Cost of follow-up treatment (THB per year)				
Outpatient follow-up	3,769 (3,015–4,523)		Gamma	e-Claim database
Inpatient costs of complications (THB per year)				
Stroke	77,379 (61,903–92,854)		Gamma	e-Claim database
Atrial fibrillation	27,841 (22,273–33,409)		Gamma	
Major bleeding	42,947 (34,358–51,537)		Gamma	
Acute kidney injury	40,175 (32,140–48,210)		Gamma	
Pacemaker implantation	292,009 (233,607–350,411)		Gamma	
Paravalvular leakage	406,704 (325,363–488,045)		Gamma	
Outpatient costs of complications (THB per year)				
Stroke	12,277 (9,821–14,732)		Gamma	e-Claim database
Atrial fibrillation	5,627 (4,501–6,752)		Gamma	
Major bleeding	5,382 (4,306–6,459)		Gamma	
Acute kidney injury	4,046 (3,237–4,856)		Gamma	
Pacemaker implantation	4,248 (3,399–5,098)		Gamma	
Paravalvular leakage	2,627 (2,102–3,153)		Gamma	
***Direct non-medical costs***				
Surgery admission (THB)				
Cost of transportation	1,295 (1,036–1,554)	1,467 (1,173–1,760)	Gamma	Patient interview
Cost of food	5,961 (4,769–7,153)	6,831 (5,465–8,197)	Gamma	
Cost of accommodation	0	1,603 (1,282–1,923)	Gamma	
Cost of informal care	20,010 (16,008–24,012)	17,795 (14,236–21,355)	Gamma	
Follow-up treatment (THB per year)				
Cost of transportation	5,108 (4,086–6,130)	5,790 (4,632–6,948)	Gamma	Patient interview
Cost of food	1,620 (1,296–1,944)	2,418 (1,935–2,902)	Gamma	
Cost of accommodation	383 (307–460)	368 (294–442)	Gamma	
Cost of informal care	11,102 (8,881–13,322)	12,086 (9,669–14,503)	Gamma	
***Utility***				
At 30-day after intervention	0.6419 (0.6290–0.6549)	0.5790 (0.5662–0.5918)	Beta	Patient interview
At 1-year after intervention	0.8276 (0.7828–0.8725)	0.8470 (0.8069–0.8871)	Beta	

Abbreviations: CAVR, conventional bioprosthetic aortic valve replacement; SUAVR, sutureless and rapid deployment aortic valve replacement; THB, Thai baht

#### Utility

At 30 days, the utility weight was estimated from the data of the NYHA functional class using the method suggested by Povero M, et al. [43] For the utility at 1 year following surgery, utility data were directly collected by interviewing patients with AS undergoing SUAVR or CAVR treatment using the Thai version of the European Quality of Life Group’s 5-dimension 5-level (EQ-5D-5L) [44]. The utility data are presented in Table 2.

### Study outcomes

The study focused on outcomes of interest such as lifetime total cost, life-years (LYs), quality-adjusted life-years (QALYs) which is the multiplication of utility and LY, incremental costs, LY gained, QALYs gained, and incremental cost-effectiveness ratio (ICER).

### Data analyses

#### Base-case analysis

In base-case analysis, the ICER was calculated in THB per LY or QALY gained by dividing the difference in total costs between SUAVR and CAVR treatments by the difference in their outcomes. In addition, the ICER was also separately calculated based on the type of AVR with or without any concomitant procedures. The costs and outcomes were considered over the lifetime horizon and were discounted annually at a rate of 3% according to the Thai HTA guideline [45]. To be considered as a cost-effective option, the estimated ICER should not exceed the Thai willingness-to-pay threshold of THB 160,000 (USD 13,652) per QALY, which is about 1.2 times per capita gross national income [46].

#### Sensitivity analyses

Both deterministic and probabilistic sensitivity analyses (PSA) were conducted to assess the uncertainty surrounding the base-case results. In the one-way sensitivity analysis, each parameter was individually varied by its specified range. In cases where specific ranges were unavailable, transitional probabilities were varied by ±10%, and costs were varied by ±20%. In addition, the discount rates for costs and outcomes were varied from 0% to 6% based on the recommendation of the Thai HTA guideline [45]. A tornado diagram was generated to exhibit the impact of input parameters variation the ICER. For PSA, the Monte Carlo simulation was iterated 1,000 times. This involved sampling all the key parameters from appropriate distributions, adhering to the guidance provided in the Thai HTA guideline [47]. Transitional probability and utility parameters were modeled using a beta distribution, while cost parameters were assigned a gamma distribution. The joint distribution of cost and QALY was plotted on the incremental cost-effectiveness plane. Moreover, a cost-effectiveness acceptability curve (CEAC) was generated to demonstrate the likelihood of SUAVR treatment being cost-effective at different levels of willingness-to-pay (WTP) values.

#### Scenario analysis

Based on the findings from an experimental study evaluating the discount rates for cost and health outcomes in the Thai context [48]. The discount rate for cost was higher than discount rate for health. The annual discount rates for cost and health outcomes were 6.2% and 1.3%, respectively. These rates differed from the recommended discount rate of 3% for both cost and health outcomes according to the Thai HTA guideline [45]. The related literature review indicated that using different discount rates in an economic evaluation could have an impact on the ICER [49]. To assess the effect of deviating from the recommended discount rates, this study applied the new discount rates to determine their impact on the estimated ICER.

## Results

### Base-case analysis

In cost-utility analysis considering patients with overall AVR from a societal perspective, SUAVR treatment incurred higher cost compared with the CAVR treatment (THB 1,733,355 [USD 147,897] vs THB 1,220,643 [USD 104,150]), while yielding lower health outcomes. SUAVR treatment resulted in 6.20 LYs and 4.95 QALYs, compared with 6.29 LYs and 5.08 QALYs for the CAVR treatment. Consequently, SUAVR treatment was dominated.

Similarly, from the healthcare system perspective, SUAVR treatment also resulted in a higher total cost compared with CAVR treatment (THB 1,594,174 [USD 136,022] vs THB 1,065,460 [USD 90,910]). Although the gains in LY and QALYs were the same as those estimated from the societal perspective, cost-utility analysis still indicated that SUAVR treatment was a dominated treatment strategy when compared with CAVR. These findings remained consistent when considering patients with isolated and combined AVR (Table 3).

**Table 3:** Base-case results.

Variables	Overall AVR		Isolated AVR		Combined AVR	
	SUAVR	CAVR	SUAVR	CAVR	SUAVR	CAVR
***Societal perspective***						
Total cost (THB/USD)	1,733,355 (147,897)	1,220,643 (104,150)	1,775,847 (151,523)	1,276,984 (108,958)	1,692,423 (144,405)	1,179,182 (100,613)
Life-years (years)	6.20	6.29	6.51	6.61	5.89	5.99
QALYs (years)	4.95	5.08	5.20	5.34	4.71	4.84
Incremental cost-effectiveness ratio						
THB/life-year (USD/life-year)	Dominated		Dominated		Dominated	
THB/QALY (USD/QALY)	Dominated		Dominated		Dominated	
***Healthcare provider perspective***						
Total cost (THB/USD)	1,594,174 (136,022)	1,065,460 (90,910)	1,631,511 (139,207)	1,116,023 (95,224)	1,557,956 (132,931)	1,029,275 (87,822)
Life-years (years)	6.20	6.29	6.51	6.61	5.89	5.99
QALYs (years)	4.95	5.08	5.20	5.34	4.71	4.84
Incremental cost-effectiveness ratio						
THB/life-year (USD/life-year)	Dominated		Dominated		Dominated	
THB/QALY (USD/QALY)	Dominated		Dominated		Dominated	

Abbreviations: AVR, aortic valve replacement; CAVR, conventional bioprosthetic aortic valve replacement; QALYs, quality-adjusted life-years; SUAVR, sutureless and rapid deployment aortic valve replacement; THB, Thai baht; USD United States dollars

Remark: Cost data were converted from Thai Baht (THB) into the United States dollars (USD) using a 2022 Purchasing Power Parity (PPP) conversion factor, as provided by the International Bank for Reconstruction and Development

### Sensitivity analyses

**Fig 2:**
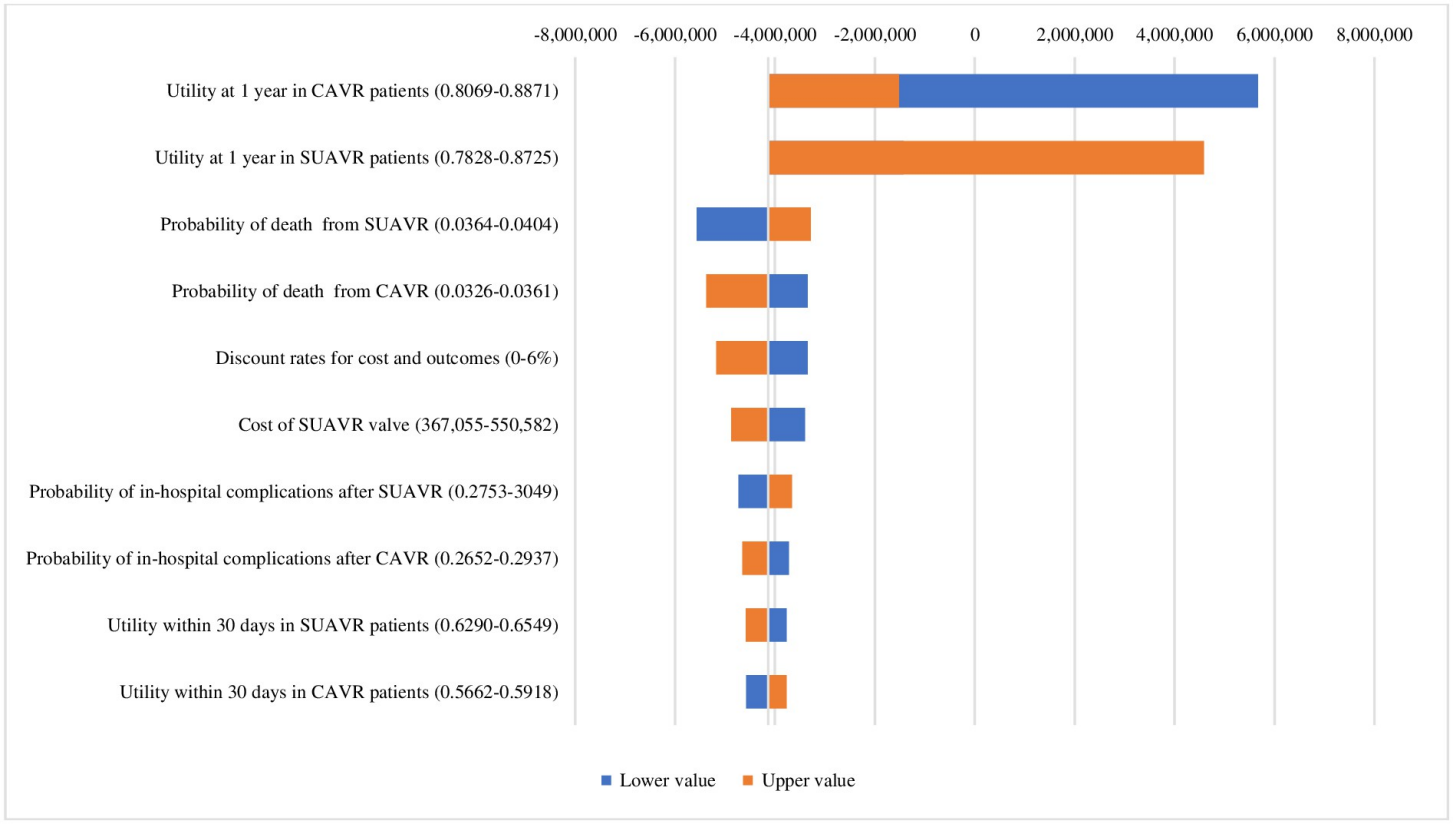
Tornado diagram of SUAVR compared with CAVR treatment. Abbreviations: CAVR, conventional bioprosthetic aortic valve replacement; SUAVR, sutureless/rapid-deployment aortic valve replacement.

The tornado diagram demonstrates the results of a cost–utility analysis from a variety of one-way sensitivity (Fig 2). The analysis revealed that the model was the most sensitive to SUAVR or CAVR treatment utilities at one year and changes in long-term mortality.

**Fig 3:**
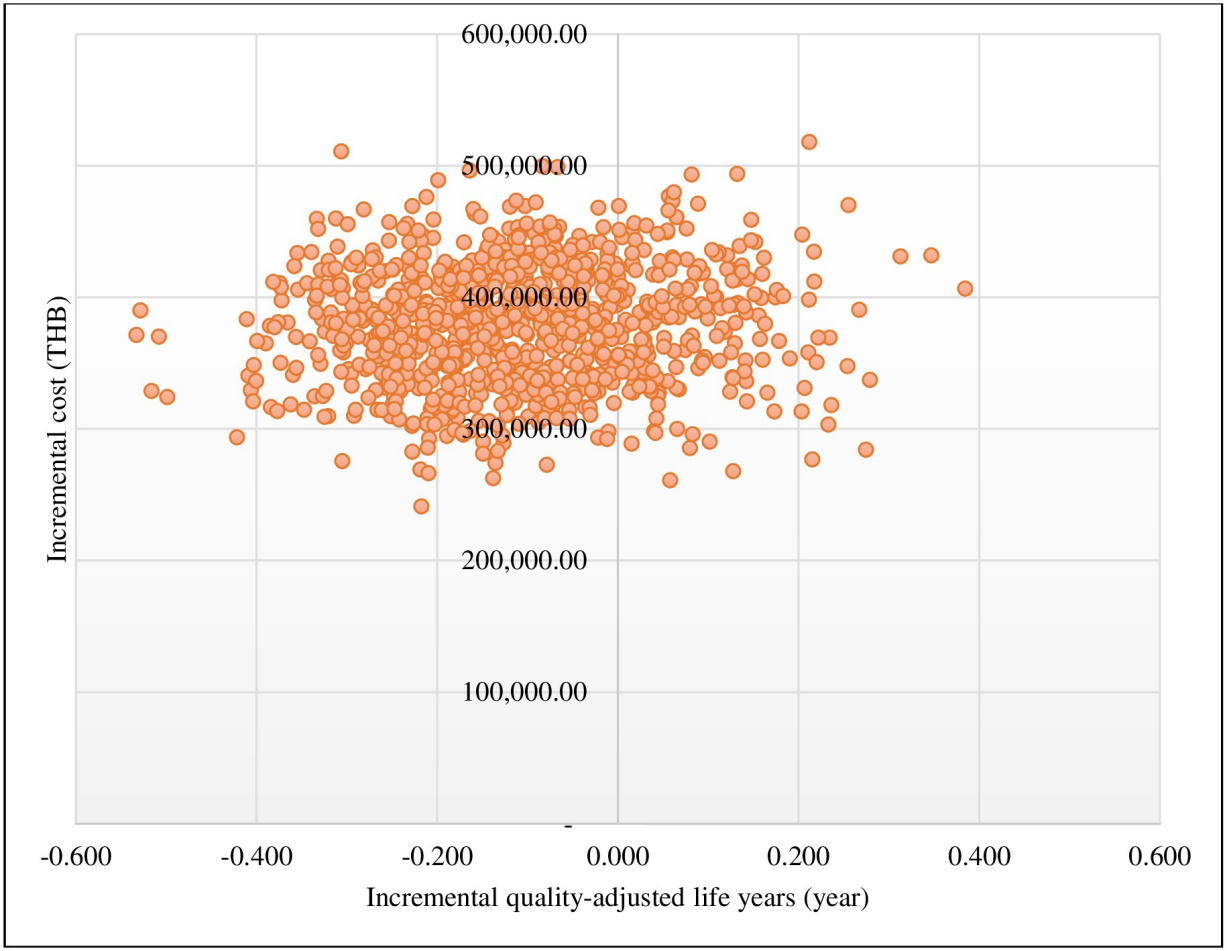
Scatter plots of 1,000 iterations for SUAVR compared with CAVR treatment on a cost-effectiveness plane. Abbreviations: CAVR, conventional bioprosthetic aortic valve replacement; SUAVR, sutureless/rapid-deployment aortic valve replacement; THB, Thai baht.

**Fig 4:**
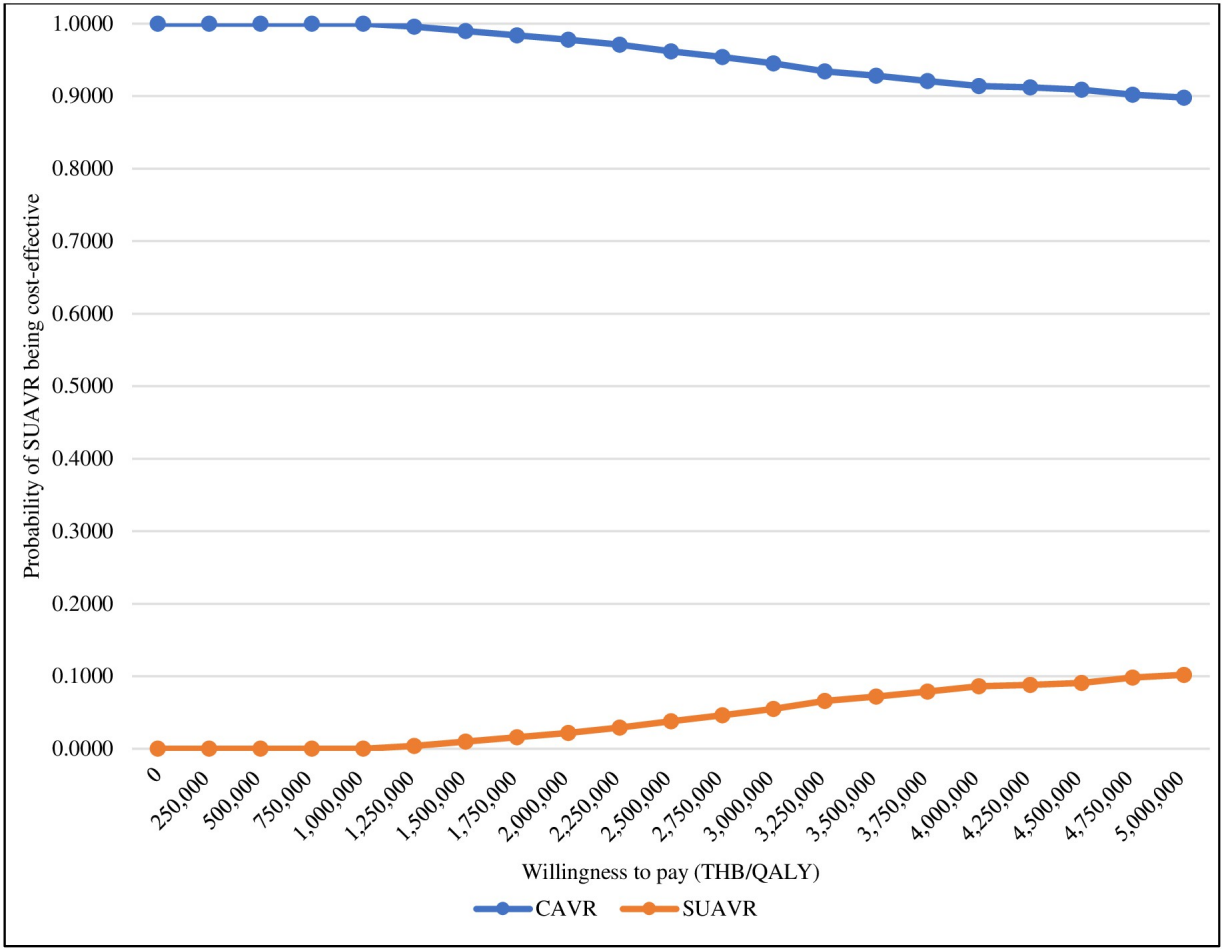
Cost-effectiveness acceptability curve of SUAVR compared with CAVR treatment. Abbreviations: CAVR, conventional bioprosthetic aortic valve replacement; QALY, quality-adjusted life-year; SUAVR, sutureless/rapid-deployment aortic valve replacement; THB, Thai baht.

The cost-effectiveness plane scatter plot showed that approximately 79% of iterations fell into the upper left quadrant (Fig 3). This indicates that SUAVR treatment was associated with higher costs and yielded fewer QALYs compared with CAVR treatment. The cost-effectiveness acceptability curve depicted the likelihood of both treatment options at various Thai WTP levels (Fig 4). CAVR treatment had a higher percentage of being cost-effective than SUAVR treatment at all levels of WTP.

### Scenario analysis

This study deviated from the recommended discount rates of 3% for cost and health outcomes and instead used new discount rates of 6.2% for costs and 1.3% for health outcomes. Consequently, the total costs of both SUAVR (THB 1,614,042 [USD 137,717]) and CAVR (THB 1,136,888 [USD 97,004]) treatments were lower compared with the base-case analysis from the societal perspective (SUAVR: THB 1,733,355 [USD 147,897] vs CAVR: THB 1,220,643 [USD 104,150]). Moreover, the health outcomes for SUAVR (6.67 LYs, 5.35 QALYs) and CAVR treatments (6.78 LYs, 5.49 QALYs) were higher than those observed in the base-case analysis (SUAVR: 6.20 LYs, 4.95 QALYs vs CAVR: 6.29 LYs, 5.08 QALYs). These findings remained consistent when considering the healthcare provider perspective (Table 4).

**Table 4:** Results of scenario analysis.

Variables	Recommended discount rate		New discount rate	
	SUAVR	CAVR	SUAVR	CAVR
***Societal perspective; Overall AVR***				
Total cost (THB/USD)	1,733,355 (147,897)	1,220,643 (104,150)	1,614,042 (137,717)	1,136,888 (97,004)
Life-years (years)	6.20	6.29	6.67	6.78
QALYs (years)	4.95	5.08	5.35	5.49
Incremental cost-effectiveness ratio				
THB/life-year (USD/life-year)	Dominated		Dominated	
THB/QALY (USD/QALY)	Dominated		Dominated	
***Healthcare provider perspective; Overall AVR***				
Total cost (THB/USD)	1,594,174 (136,022)	1,065,460 (90,910)	1,488,424 (126,998)	997,549 (85,115)
Life-years (years)	6.20	6.29	6.67	6.78
QALYs (years)	4.95	5.08	5.35	5.49
Incremental cost-effectiveness ratio				
THB/life-year (USD/life-year)	Dominated		Dominated	
THB/QALY (USD/QALY)	Dominated		Dominated	

Abbreviations: AVR, aortic valve replacement; CAVR, conventional bioprosthetic aortic valve replacement; QALYs, quality-adjusted life-years; SUAVR, sutureless and rapid deployment aortic valve replacement; THB, Thai baht; USD United States dollars

Remark: Cost data were converted from Thai Baht (THB) into the United States dollars (USD) using a 2022 Purchasing Power Parity (PPP) conversion factor, as provided by the International Bank for Reconstruction and Development

## Discussion

This study constitutes the first health economic evaluation using local available cost and utility data to compare the cost-utility of SUAVR vs. CAVR treatment in patients with moderate to severe AS in Thailand. Based on the findings of this study, the estimated ICER indicated that SUAVR treatment was dominated, resulting in SUAVR treatment not being a cost-effective treatment, compared with CAVR treatment.

Three related cost-effectiveness studies conducted in the US [10, 11] and Norway [9] reported that SUAVR treatment was a cost-effective strategy, compared with CAVR treatment in patient with AS. In the US, SUAVR treatment was found to be a dominant strategy when compared with CAVR treatment, regardless of whether minimally invasive surgery (MIS) [10] or full-sternotomy (FS) procedures [11] were performed. Similarly, in Norway, SUAVR treatment was found as a dominant strategy when compared with CAVR treatment across types of surgical procedures, including FS, MIS, and concomitant procedures [9]. The findings from both countries indicated that SUAVR had lower total cost and higher LYs or QALYs than CAVR treatment. However, the findings of this study were not in line with the findings from the US and Norway due to several reasons. Firstly, SUAVR treatment shows obvious benefits in terms of the reduced hospitalization and operation times like ACC and CPB times. This benefit was not clearly shown in terms of cost reduction in Thailand. The method to estimate operation costs in this study was the gross costing approach; therefore, the operation cost was capitated although the operative time was shortened. We encourage the future cost-effectiveness study to use a micro-costing approach to capture the cost reduction from benefits accrued by the SUAVR treatment. Secondly, the cost of valves significantly differed among countries. We found that the additional cost incurred by the SUAVR valve was about 3.3 times or THB 321,632 (USD 27,443) higher than the cost of a conventional bioprosthetic valve. However, the magnitude difference of the valve cost in the US was about USD 6,000 [10, 11], and 2.8 times in Norway [9]. Thirdly, the acceptable threshold in Thailand is much lower than that in those countries. This leads to a smaller opportunity of new health technology being accepted in Thailand. Fourthly, long-term outcome data in the Markov model were unavailable in Thailand. The results from short-term meta-analysis indicated a lower mortality rate of SUAVR treatment compared with CAVR treatment (RR 0.97, 95% CI 0.84–1.12). When the RR was applied to the mortality risk of CAVR treatment, the mortality risk of SUAVR became lower. This indicated a prolonged life expectancy of patients undergoing SUAVR treatment compared with patients undergoing CAVR treatment. For the long-term outcomes in the Markov model, the mortality risks of both treatments were pooled from the two RCTs [15, 17] and a propensity-score matched observational study [36], showing the opposite direction from the short-term outcome. We found that CAVR treatment exhibited a lower mortality risk than the SUAVR treatment. As a consequence, LYs of SUAVR treatment in this study were shorter than those of CAVR treatment. Finally, the utility value at one year after CAVR treatment was slightly higher than that after SUAVR treatment. This resulted in decrease QALYs gained in SUAVR treatment compared with those of CAVR treatment.

In the scenario analysis, the recommendation of applying a discount rate of 3% for both costs and health outcomes based on the Thai HTA guideline [45] was established before the Coronavirus disease 2019 (COVID-19) pandemic. These rates may not accurately reflect the current economic conditions in Thailand. Therefore, a valid concern remains regarding the appropriateness of these recommended rates. To address this potential threat, our previous experimental study was designed to determine the appropriate degree of discounting and consider whether equal discounting should be applied to both costs and health outcomes. This investigation resulted in determining annual discount rates for costs and health outcomes, which were estimated at 6.2% and 1.3%, respectively [48]. In the present study, we applied these newly derived discount rates as part of a scenario analysis. The results of this analysis demonstrated that the using these derived discount rates led to increased health outcomes measured in terms of LYs and reduced costs when compared with the recommended discount rates. Consequently, this scenario analysis highlights the potential long-term advantages associated with a health intervention and enhances the likelihood of it being considered as a cost-effectiveness strategy. These findings could serve as a case study to support the rationale for justifying appropriate discount rates for both costs and health outcomes in the context of health economic evaluation in Thailand.

This cost-utility analysis relied on Thai-specific cost and utility data, obtained directly from patients with moderate to severe AS undergoing either SUAVR or CAVR treatments at three large hospitals. Our study encountered some limitations. Firstly, this study initiated data collection during the COVID-19 outbreak. A few new cases were enrolled for data collection, especially utility value during the short-term period. We addressed this limitation by estimating utility value from the NYHA status instead. However, it might not be as accurate as direct patient-reported outcomes. However, it is acknowledged that this indirect approach may not be as precise as directly obtaining patient-reported outcomes. Secondly, another limitation was regarding the transitional probabilities used in the long-term Markov model. We derived the transitional probabilities by pooling the data from two RCTs and a propensity-score matched cohort study at one year, and then carried forward the constant transitional probabilities. This could potentially introduce some uncertainty into the study’s results. Thirdly, regarding the cost of intervention, we collected data from three well-known hospitals, comprising two university-affiliated hospitals and a specialized medical institute. These institutions significantly contribute to representing the Thai population undergoing aortic valve replacement, whether using sutureless or conventional valves. Thus, this data source is deemed reliable for our study. However, we acknowledge that the incorporation of hospitals mainly situated in Bangkok and its surrounding areas might restrict the generalizability of our findings to the wider Thai healthcare system, given the absence of data from regional or local areas. Finally, the utilization of Thai-specific cost and utility data in the model represents a notable strength within the context of the Thai healthcare system. However, it may be perceived as a limitation when extrapolating the findings to other settings.

This study was requested by the SCBP in order to generate cost-effectiveness evidence for policy makers to justify the SUAVR treatment into the UHCBP under the UHCS in Thailand. While the study results indicate that SUAVR treatment does not meet the criteria for cost-effectiveness, several important issues were identified through interviews and focus-group discussions with cardiothoracic surgeons and nurses. Firstly, recognizing that SUAVR treatment may be deemed necessary for specific patient groups is essential. This includes patients with AS undergoing AVR through minimally invasive surgery, those undergoing AVR with concomitant procedures, and patients with calcified and small aortic root. Secondly, despite the SUAVR valve being a novel medical device, it is noteworthy that the surgical techniques and practices employed by cardiothoracic surgeons and nurses do not differ significantly from those used with conventional bioprosthetic valves. Moreover, the existing healthcare facilities, including medical equipment, staffing, and the referral system within tertiary or university-affiliated hospitals in Thailand, are sufficient to support the policy of SUAVR reimbursement. Therefore, financial investment in terms of building new facilities to accommodate SUAVR treatment is not required.

## Conclusion

Our findings indicated that SUAVR is not a cost-effective strategy compared with CAVR for patients with moderate to severe AS in Thailand, as it leads to higher costs and inferior health outcomes from both societal and healthcare provider perspectives. Other important issues related to specific patients such as those with minimally invasive surgery, those undergoing AVR with concomitant procedures, and those with calcified and small aortic root should be taken into account.

## Supporting information

S1 File (PDF)

## References

[1] Osnabrugge RL, Mylotte D, Head SJ, Van Mieghem NM, Nkomo VT, LeReun CM, et al. Aortic stenosis in the elderly: disease prevalence and number of candidates for transcatheter aortic valve replacement: a meta-analysis and modeling study. J Am Coll Cardiol. 2013;62(11):1002–12 doi: 10.1016/j.jacc.2013.05.015 23727214

[2] Turina J, Hess O, Sepulcri F, Krayenbuehl HP. Spontaneous course of aortic valve disease. Eur Heart J. 1987;8(5):471–83 doi: 10.1093/oxfordjournals.eurheartj.a062307 3609042

[3] Vahanian A, Beyersdorf F, Praz F, Milojevic M, Baldus S, Bauersachs J, et al. 2021 ESC/EACTS Guidelines for the management of valvular heart disease. Eur Heart J. 2022;43(7):561–632 doi: 10.1093/eurheartj/ehab395 34453165

[4] Otto CM, Nishimura RA, Bonow RO, Carabello BA, Erwin JP 3rd, Gentile F, et al. 2020 ACC/AHA Guideline for the Management of Patients With Valvular Heart Disease: Executive Summary: A Report of the American College of Cardiology/American Heart Association Joint Committee on Clinical Practice Guidelines. Circulation. 2021;143(5):e35–e71 doi: 10.1161/CIR.0000000000000932 33332149

[5] Gersak B, Fischlein T, Folliguet TA, Meuris B, Teoh KH, Moten SC, et al. Sutureless, rapid deployment valves and stented bioprosthesis in aortic valve replacement: recommendations of an International Expert Consensus Panel. Eur J Cardiothorac Surg. 2016;49(3):709–18 doi: 10.1093/ejcts/ezv369 26516193

[6] Glauber M, Moten SC, Quaini E, Solinas M, Folliguet TA, Meuris B, et al. International Expert Consensus on Sutureless and Rapid Deployment Valves in Aortic Valve Replacement Using Minimally Invasive Approaches. Innovations (Phila). 2016;11(3):165–73 doi: 10.1097/IMI.0000000000000287 27540996 PMC4996354

[7] Erfe JM, Malaisrie SC, Andrei AC, Pham DT, Churyla A, Kruse J, et al. Outcomes of Sutureless/Rapid Deployment Valves Compared to Traditional Bioprosthetic Aortic Valves. Ann Thorac Surg. 2021;111(6):1884–91 doi: 10.1016/j.athoracsur.2020.07.034 32987022

[8] D’Onofrio A, Salizzoni S, Filippini C, Tessari C, Bagozzi L, Messina A, et al. Surgical aortic valve replacement with new-generation bioprostheses: Sutureless versus rapid-deployment. J Thorac Cardiovasc Surg. 2020;159(2):432–42.e1 doi: 10.1016/j.jtcvs.2019.02.135 31213376

[9] Desser AS, Arentz-Hansen H, Fagerlund BF, Harboe I, Lauvrak V. Sutureless Aortic Valve Replacement for Treatment of Severe Aortic Stenosis: A Single Technology Assessment of Perceval Sutureless Aortic Valve. Oslo, Norway: Knowledge Centre for the Health Services at The Norwegian Institute of Public Health (NIPH); 2017. 29553663

[10] Moore M, Barnhart GR, Chitwood WR Jr., Rizzo JA, Gunnarsson C, Palli SR, et al. The economic value of INTUITY in aortic valve replacement. J Med Econ. 2016;19(10):1011–7 doi: 10.1080/13696998.2016.1220949 27549435

[11] Moore M, Barnhart GR, Chitwood WR Jr., Rizzo JA, Gunnarsson C, Palli SR, et al. The economic value of rapid deployment aortic valve replacement via full sternotomy. J Comp Eff Res. 2017;6(4):293–302 doi: 10.2217/cer-2016-0064 28374618

[12] Jonathan ACS, Jelena S, Matthew JP, Roy GE, Natalie SB, Isabelle B, et al. RoB 2: a revised tool for assessing risk of bias in randomised trials. BMJ. 2019;366:l4898 doi: 10.1136/bmj.l4898 31462531

[13] ROBINS-E Development Group. Risk Of Bias In Non-randomized Studies—of Exposure (ROBINS-E). Launch version, 1 June 2022. 2022 [https://www.riskofbias.info/welcome/robins-e-tool.

[14] Borger MA, Moustafine V, Conradi L, Knosalla C, Richter M, Merk DR, et al. A randomized multicenter trial of minimally invasive rapid deployment versus conventional full sternotomy aortic valve replacement. Ann Thorac Surg. 2015;99(1):17–25 doi: 10.1016/j.athoracsur.2014.09.022 25441065

[15] Borger MA, Dohmen PM, Knosalla C, Hammerschmidt R, Merk DR, Richter M, et al. Haemodynamic benefits of rapid deployment aortic valve replacement via a minimally invasive approach: 1-year results of a prospective multicentre randomized controlled trial. Eur J Cardiothorac Surg. 2016;50(4):713–20 doi: 10.1093/ejcts/ezw042 26935407

[16] Dedeilias P, Baikoussis NG, Prappa E, Asvestas D, Argiriou M, Charitos C. Aortic valve replacement in elderly with small aortic root and low body surface area; the Perceval S valve and its impact in effective orifice area. J Cardiothorac Surg. 2016;11(1):54 doi: 10.1186/s13019-016-0438-7 27066903 PMC4827171

[17] Fischlein T, Folliguet T, Meuris B, Shrestha ML, Roselli EE, McGlothlin A, et al. Sutureless versus conventional bioprostheses for aortic valve replacement in severe symptomatic aortic valve stenosis. J Thorac Cardiovasc Surg. 2021;161(3):920–32 doi: 10.1016/j.jtcvs.2020.11.162 33478837

[18] Gilmanov D, Miceli A, Ferrarini M, Farneti P, Murzi M, Solinas M, et al. Aortic valve replacement through right anterior minithoracotomy: can sutureless technology improve clinical outcomes? Ann Thorac Surg. 2014;98(5):1585–92 doi: 10.1016/j.athoracsur.2014.05.092 25200732

[19] Pollari F, Santarpino G, Dell’Aquila AM, Gazdag L, Alnahas H, Vogt F, et al. Better short-term outcome by using sutureless valves: a propensity-matched score analysis. Ann Thorac Surg. 2014;98(2):611–6; discussion 6–7 doi: 10.1016/j.athoracsur.2014.04.072 24928678

[20] Dalén M, Biancari F, Rubino AS, Santarpino G, De Praetere H, Kasama K, et al. Ministernotomy versus full sternotomy aortic valve replacement with a sutureless bioprosthesis: a multicenter study. Ann Thorac Surg. 2015;99(2):524–30 doi: 10.1016/j.athoracsur.2014.08.028 25483001

[21] Muneretto C, Alfieri O, Cesana BM, Bisleri G, De Bonis M, Di Bartolomeo R, et al. A comparison of conventional surgery, transcatheter aortic valve replacement, and sutureless valves in "real-world" patients with aortic stenosis and intermediate- to high-risk profile. J Thorac Cardiovasc Surg. 2015;150(6):1570–7; discussion 7–9 doi: 10.1016/j.jtcvs.2015.08.052 26384753

[22] Forcillo J, Bouchard D, Nguyen A, Perrault L, Cartier R, Pellerin M, et al. Perioperative outcomes with sutureless versus stented biological aortic valves in elderly persons. J Thorac Cardiovasc Surg. 2016;151(6):1629–36 doi: 10.1016/j.jtcvs.2015.12.056 26896213

[23] Smith AL, Shi WY, Rosalion A, Yii M, O’Keefe M, Newcomb AE, et al. Rapid-Deployment Versus Conventional Bio-Prosthetic Aortic Valve Replacement. Heart Lung Circ. 2017;26(2):187–93 doi: 10.1016/j.hlc.2016.06.1202 27523460

[24] Ensminger S, Fujita B, Bauer T, Möllmann H, Beckmann A, Bekeredjian R, et al. Rapid Deployment Versus Conventional Bioprosthetic Valve Replacement for Aortic Stenosis. J Am Coll Cardiol. 2018;71(13):1417–28 doi: 10.1016/j.jacc.2018.01.065 29598861

[25] Nguyen A, Stevens LM, Bouchard D, Demers P, Perrault LP, Carrier M. Early Outcomes with Rapid-deployment vs Stented Biological Valves: A Propensity-match Analysis. Semin Thorac Cardiovasc Surg. 2018;30(1):16–23 doi: 10.1053/j.semtcvs.2017.09.002 29031706

[26] Rahmanian PB, Kaya S, Eghbalzadeh K, Menghesha H, Madershahian N, Wahlers T. Rapid Deployment Aortic Valve Replacement: Excellent Results and Increased Effective Orifice Areas. Ann Thorac Surg. 2018;105(1):24–30 doi: 10.1016/j.athoracsur.2017.07.047 29132703

[27] Repossini A, Fischlein T, Solinas M, Di Bacco L, Passaretti B, Grubitzsch H, et al. Stentless sutureless and transcatheter valves: a comparison of the hemodynamic performance of different prostheses concept. Minerva Cardioangiologica. 2018;66(2):180–90 doi: 10.23736/S0026-4725.17.04564-9 29160045

[28] Rubino AS, Santarpino G, De Praetere H, Kasama K, Dalén M, Sartipy U, et al. Early and intermediate outcome after aortic valve replacement with a sutureless bioprosthesis: Results of a multicenter study. J Thorac Cardiovasc Surg. 2014;148(3):865–71; discussion 71 doi: 10.1016/j.jtcvs.2014.03.052 24954175

[29] Gotzmann M, Wilbring M, Charitos E, Treede H, Silaschi M. Hemodynamic Comparison of Sutureless and Rapid-Deployment Valves with Conventional Bioprostheses. Thorac Cardiovasc Surg. 2020;68(7):584–94 doi: 10.1055/s-0039-1683426 30900219

[30] Hartrumpf M, Kuehnel RU, Schroeter F, Haase R, Laux ML, Ostovar R, et al. Clinical Short-Term Outcome and Hemodynamic Comparison of Six Contemporary Bovine Aortic Valve Prostheses. Thorac Cardiovasc Surg. 2020;68(7):557–66 doi: 10.1055/s-0038-1676853 30669172

[31] Herry M, Laghlam D, Touboul O, Nguyen LS, Estagnasié P, Brusset A, et al. Pacemaker implantation after aortic valve replacement: rapid-deployment Intuity^®^ compared to conventional bioprostheses. Eur J Cardiothorac Surg. 2020;58(2):335–42 doi: 10.1093/ejcts/ezaa068 32215660 PMC7373323

[32] Choi JW, Kim HJ, Kim JB, Lee S, Lim C, Chang BC, et al. Early and Two-year Outcomes after Sutureless and Conventional Aortic Valve Replacement: a Nationwide Population-based Study. J Korean Med Sci. 2021;36(9):e57 doi: 10.3346/jkms.2021.36.e57 33686809 PMC7940122

[33] Paparella D, Santarpino G, Moscarelli M, Guida P, De Santis A, Fattouch K, et al. Minimally invasive aortic valve replacement: short-term efficacy of sutureless compared with stented bioprostheses. Interact Cardiovasc Thorac Surg. 2021;33(2):188–94 doi: 10.1093/icvts/ivab070 33984125 PMC8691673

[34] Berretta P, Andreas M, Meuris B, Langenaeken T, Solinas M, Concistrè G, et al. Sutureless and rapid deployment versus sutured aortic valve replacement: a propensity-matched comparison from the Sutureless and Rapid Deployment International Registry. Eur J Cardiothorac Surg. 2022;62(2) doi: 10.1093/ejcts/ezac378 35775935

[35] Bottio T, Piperata A, Guariento A, Lorenzoni G, Cavicchiolo AG, Gemelli M, et al. Standard versus rapid-deployment aortic valve replacement and concomitant myocardial revascularization: 5-year bi-centre clinical outcomes. Eur J Cardiothorac Surg. 2022;62(5) doi: 10.1093/ejcts/ezac476 36190347

[36] Ono Y, Yajima S, Kainuma S, Kawamoto N, Tadokoro N, Kakuta T, et al. Early Outcomes of Intuity Rapid Deployment Aortic Valve Replacement Compared With Conventional Biological Valves in Japanese Patients. Circ J. 2022;86(11):1710–8 doi: 10.1253/circj.CJ-21-0959 35569971

[37] Santarpino G, Lorusso R, Peivandi AD, Atzeni F, Avolio M, Dell’Aquila AM, et al. In-Hospital Mortality and Risk Prediction in Minimally Invasive Sutureless versus Conventional Aortic Valve Replacement. Journal of Clinical Medicine. 2022;11(24) doi: 10.3390/jcm11247273 36555892 PMC9783653

[38] D’Onofrio A, Cibin G, Lorenzoni G, Tessari C, Bifulco O, Lombardi V, et al. Propensity-Weighted Comparison of Conventional Stented and Rapid-Deployment Aortic Bioprostheses. Curr Probl Cardiol. 2023;48(1):101426 doi: 10.1016/j.cpcardiol.2022.101426 36181783

[39] World Health Organization. Global Health Observatory data repository: Life tables by country (Thailand) 2022 [https://apps.who.int/gho/data/?theme=main&vid=61640.

[40] Riewpaiboon A. Measurement of costs for health economic evaluation. Journal of the Medical Association of Thailand = Chotmaihet thangphaet. 2014;97 Suppl 5:S17–26 24964695

[41] Bureau of Trade and Economics Indices, Ministry of Commerce. Consumer Price Index 2022 [Cited 30 March 2023]. http://www.price.moc.go.th/price/cpi/index_new.asp.

[42] The International Bank for Reconstruction and Development. PPP conversion factor, GDP (LCU per international $) 2022 [https://data.worldbank.org/indicator/PA.NUS.PPP.

[43] Povero M, Miceli A, Pradelli L, Ferrarini M, Pinciroli M, Glauber M. Cost-utility of surgical sutureless bioprostheses vs TAVI in aortic valve replacement for patients at intermediate and high surgical risk. Clinicoecon Outcomes Res. 2018;10:733–45 doi: 10.2147/CEOR.S185743 30510436 PMC6231515

[44] Pattanaphesaj J. Health-related quality of life measure (EQ-5D-5L): measurement property testing and its preference-based score in Thai population [Doctoral dissertation]. Bangkok: Mahidol University; 2014.

[45] Permsuwan U, Guntawongwan K, Buddhawongsa P. Handling time in economic evaluation studies. Journal of the Medical Association of Thailand = Chotmaihet thangphaet. 2014;97 Suppl 5:S50–8 24964699

[46] Thavorncharoensap M, Teerawattananon Y, Natanant S, Kulpeng W, Yothasamut J, Werayingyong P. Estimating the willingness to pay for a quality-adjusted life year in Thailand: does the context of health gain matter? Clinicoecon Outcomes Res. 2013;5:29–36 doi: 10.2147/CEOR.S38062 23345984 PMC3548562

[47] Limwattananon S. Handling uncertainty of the economic evaluation result: sensitivity analysis. Journal of the Medical Association of Thailand = Chotmaihet thangphaet. 2008;91 Suppl 2:S59–65 19253488

[48] Yadee J, Permsuwan U, Guntawongwan K, Himakalasa W, Buddhawongsa P. Discounting money and health effects from communicable and noncommunicable diseases in Thailand. Sci Rep. 2023;13(1):3324 doi: 10.1038/s41598-023-30559-2 36849620 PMC9969024

[49] Bos JM, Postma MJ, Annemans L. Discounting health effects in pharmacoeconomic evaluations: current controversies. Pharmacoeconomics. 2005;23(7):639–49 doi: 10.2165/00019053-200523070-00001 16173156

