# Supplementary material for: Cost-utility analysis of sutureless and rapid deployment versus conventional aortic valve replacement in patients with moderate to severe aortic stenosis in Thailand
Source: PLoS One. 2024 Jan 19;19(1):e0296875. doi: 10.1371/journal.pone.0296875 (PMC10798510; doi:10.1371/journal.pone.0296875)
Supplement: S1 File — (PDF) [file pone.0296875.s001.pdf]

Supplement materials

Cost-utility Analysis of Sutureless and Rapid Deployment versus Conventional Aortic Valve Replacement in Patients with Moderate to Severe Aortic Stenosis in Thailand

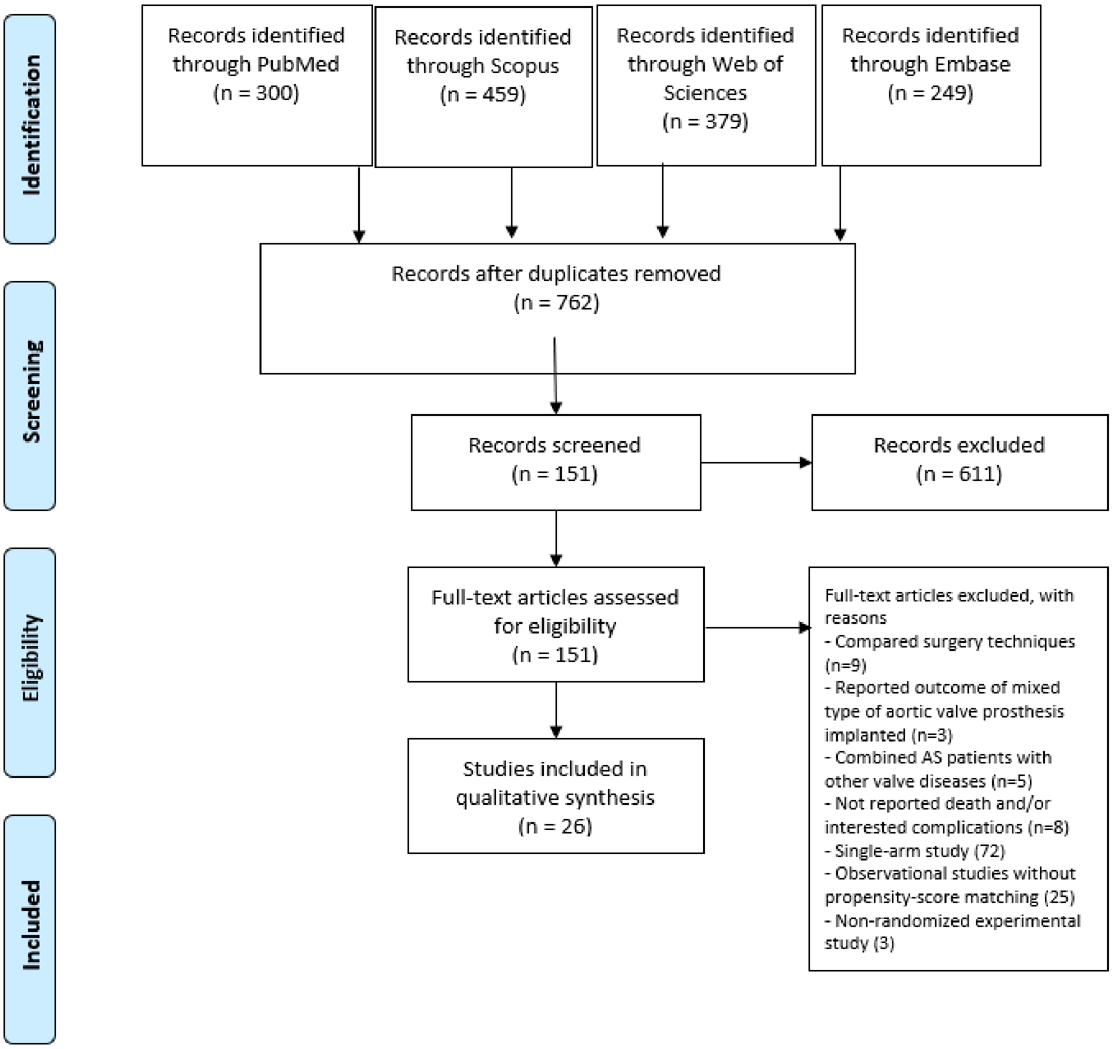

Number of included studies		
**Outcome**	**Randomized controlled trial**	**Propensity-score matched cohort study**
Short term (30 days)	4 [1-4]	22 [5-26]
Long term (1 year)	2 [2, 4]	1 [24]

Figure S1 Flow diagram of study selection

Table S1 Search strategies

Period: from inception to December 2022

Search number	Query	Results
**Medline via PubMed**		
1	"aortic valve" [mh] OR “aortic valve” [tiab] OR "aortic valve replacement" [tiab] OR "aortic valve prosthesis" [tiab] OR "aortic prosthesis" [tiab] OR “prosthetic aortic valve” [tiab] OR “aortic valve implantation” [tiab]	66,155
2	"sutureless surgical procedures" [mh] OR ‘‘sutureless’’ [tiab] OR ‘‘rapid deployment’’ [tiab] OR “rapid-deployment” [tiab]	3,878
3	Perceval* [tiab] OR “3F Enable” [tiab] OR 3F-enable [tiab] OR intuity [tiab]	462
4	#1 AND (#2 OR #3)	817
5	“randomized controlled trial” [pt] OR “controlled clinical trial” [pt] OR randomized [mh] OR randomized* [tiab] OR randomised [mh] OR randomised* [tiab] OR clinical trials [majr] OR “clinical trials” [tiab] OR randomly [tiab] OR trial* [ti] OR placebo* [tiab]	1,717,944
6	efficacy [tiab] OR effectiveness [tiab] OR outcome* [tiab] OR "perioperative outcome*" [tiab] OR “peri-operative outcome*” [tiab] OR “postoperative outcome*” [tiab] OR “post-operative outcome*” [tiab]	3,410,371
7	“cross-clamp time” [tiab] OR “cardiopulmonary bypass time” [tiab] OR “operation time” [tiab]	22,122
8	(“intensive care unit” [tiab] OR “non-intensive care unit” [tiab] OR ward [tiab] OR hospital [tiab]) AND (“length of stay” [tiab] OR stay [tiab])	161,621
9	#5 OR #6 OR #7 OR #8	4,536,897
10	#4 AND #9	367
11	Limit #10 to Human	300
**Scopus**		
1	TITLE-ABS-KEY ("aortic valve" OR "aortic valve replacement" OR "aortic valve prosthesis" OR "aortic prosthesis" OR “prosthetic aortic valve” OR “aortic valve implantation”)	92,119
2	TITLE-ABS-KEY (sutureless OR "rapid deployment" OR rapid-deployment)	6,732
3	TITLE-ABS-KEY (Perceval* OR “3F Enable” OR 3F-enable OR intuity)	918
4	#1 AND (#2 OR #3)	1,009
5	TITLE-ABS-KEY ("randomized controlled trial" OR "controlled clinical trial" OR randomized OR randomized* OR randomized OR randomised* OR "clinical trials" OR randomly OR trial* OR placebo*)	3,556,492
**6**	TITLE-ABS-KEY **(**efficacy OR effectiveness OR outcome* OR "perioperative outcome*"OR “peri-operative outcome*” OR “postoperative outcome*” OR “post-operative outcome*”)	7,317,907
7	TITLE-ABS-KEY (“cross-clamp time” OR “cardiopulmonary bypass time” OR “operation time”)	38,277
8	TITLE-ABS-KEY (("intensive care unit" OR "non-intensive care unit" OR ward OR hospital) AND ("length of stay" OR stay))	264,100
9	#5 OR #6 OR #7 OR #8	9,534,559
10	#4 AND #11	689
11	Limit #12 to Human, Journal article	459
**Web of Sciences**		
1	TS = ("aortic valve" OR "aortic valve replacement" OR "aortic valve prosthesis" OR "aortic prosthesis" OR “prosthetic aortic valve” OR “aortic valve implantation”)	59,629
2	TS = (sutureless* OR "rapid deployment" OR rapid-deployment)	5,749
3	TS = (Perceval* OR “3F Enable” OR 3F-enable OR intuity)	806
4	#1 AND (#2 OR #3)	838

5	TS = (“randomized controlled trial*” OR “controlled clinical trial*” OR randomized* OR randomised* OR placebo* OR randomly*) OR TI = (“clinical trials*” OR trial)	1,797,435
6	TS = (efficacy OR effectiveness OR outcome* OR "perioperative outcome*"OR “peri-operative outcome*” OR “postoperative outcome*” OR “post-operative outcome*”)	5,161,835
7	TS = (“cross-clamp time” OR “cardiopulmonary bypass time” OR “operation time”)	24,081
8	TS = (“intensive care unit” OR “non-intensive care unit” OR ward OR hospital) AND TS = (“length of stay” OR stay)	166,229
9	#5 OR #6 OR #7 OR #8	6,351,176
10	#4 AND #11	481
11	#4 AND #11 and Articles (Document Types)	379
**Embase**		
1	'aortic valve'/exp OR 'aortic valve':ab,ti OR 'aortic valve replacement'/exp OR 'aortic valve replacement':ab,ti OR 'aortic valve prosthesis'/exp OR 'aortic valve prosthesis':ab,ti OR 'aortic prosthesis'/exp OR 'aortic prosthesis':ab,ti OR 'prosthetic aortic valve':ab,ti OR 'aortic valve implantation':ab,ti	106,157
2	'sutureless technique'/exp OR sutureless:ab,ti OR 'rapid deployment aortic valve replacement'/exp OR 'rapid deployment;ab,ti'	4,136
3	perceval*:ab,ti OR '3f enable':ab,ti OR '3f enable;ab,ti' OR intuity:ab,ti	717
4	#1 AND (#2 OR #3)	1,172
5	'randomized controlled trial':ab,ti OR 'controlled clinical trial':ab,ti OR randomized:ab,ti OR randomized*:ab,ti OR randomised:ab,ti OR randomised*:ab,ti OR 'clinical trials':ab,ti OR randomly:ab,ti OR trial*:ab,ti OR placebo:ab,ti	2,604,596
6	efficacy:ab,ti OR effectiveness:ab,ti OR 'outcome*':ab,ti OR 'perioperative outcome*':ab,ti OR 'peri-operative outcome*':ab,ti OR 'postoperative outcome*':ab,ti OR 'post-operative outcome*':ab,ti	4,889,542
7	‘cross-clamp time’:ab,ti OR ‘cardiopulmonary bypass time’:ab,ti OR ‘operation time’:ab,ti	34,369
8	(‘intensive care unit’:ab,ti OR ‘non-intensive care unit’:ab,ti OR ward:ab,ti OR hospital:ab,ti) AND (‘length of stay’:ab,ti OR stay:ab,ti)	278,204
9	#5 OR #6 OR #7 OR #8	6,517,754
10	#4 AND #9	584
11	#12 AND 'human'/de AND 'article'/it	249

Table S2 Characteristics of included studies

No	First authors	Year of publication	Site/country	Recruitment period	No. of Patients		Devices		Outcome reported	
					SUAVR	CAVR	SUAVR	CAVR	30-day	1-year
**Randomized controlled trials**										
1	Borger MA [1]	2015	Germany	2012-2013	51	49	Edwards Intuity	Bioprosthetic valve (Hancock II, Mitroflow, Trifecta, Perimount Magna Ease)	Y	
2	Borger MA [2]	2016	Germany	2012-2013	46	48	Edwards Intuity	Bioprosthetic valve (Hancock II, Mitroflow, Trifecta, Perimount Magna Ease)	Y	Y
3	Dedeilias P [3]	2016	Greece	2012-2014	25	25	Perceval S	Bioprosthetic valve (Soprano)	Y	
4	Fischlein T [4]	2021	Austria, Belgium, Canada, Chile, France, Germany, Israel, Italy, Spain, The Netherlands, The United Kingdom, TheUnited States	2016-2018	453	457	Perceval S	Bioprosthetic valve	Y	Y
**Propensity-score matched observational studies**										
5	Gilmanov D [5]	2014	Italy	2004-2014	133	133	Perceval S (125) / Edwards Intuity (6) / 3f Enable (2)	Bioprosthetic valve (Perimount, Mosaic)	Y	
6	Pollari F [6]	2014	Germany	2010-2013	166	400	Perceval S	Bioprosthetic valve	Y	
7	Dalén M [7]	2015	Belgium, Finland, Germany, Italy, Sweden	2007- 2014	171	171	Perceval S	Bioprosthetic valve (Perimount)	Y	
8	Muneretto C [8]	2015	Italy	2007-2014	204	204	Perceval S	Bioprosthetic valve (Perimount Magna Ease,Trifecta , Mitroflow, Freedom Solo)	Y	
9	Forcillo J [9]	2016	Canada	2011- 2015	65	130	Perceval S	Bioprosthetic valve (Perimount, Magna Ease, Mosaic, Mitroflow, Biocor, Epic Standard, Epic Supra)	Y	
10	Smith AL [10]	2017	Australia, New Zealand	2008-2015	41	41	Edwards Intuity / 3f Enable	Bioprosthetic valve	Y	
11	Ensminger S [11]	2018	Germany	2011-2015	1,021	1,021	Perceval S / Edwards Intuity / 3f Enable	Bioprosthetic valve	Y	

No	First authors	Year of publication	Site/country	Recruitment period	No. of Patients		Devices		Outcome reported	
					SUAVR	CAVR	SUAVR	CAVR	30-day	1-year
12	Nguyen A [12]	2018	Canada	2012-2015	59	177	Edwards Intuity	Bioprosthetic valve (Magna Ease, Perimount, Mosaic, Mitroflow, Trifecta)	Y	
13	Rahmanian PB [13]	2018	Germany	2011-2017	163	163	Edwards Intuity	Bioprosthetic valve (Perimount)	Y	
14	Repossini A [14]	2018	Italy, Germany,France	2010-2016	185	185	Perceval S	Bioprosthetic valve (Freedom Solo)	Y	
15	Rubino AS [15]	2018	Italy	2011-2015	39	39	Perceval S	Bioprosthetic valve	Y	
16	Gotzmann M [16]	2020	Germany	2016-2017 (SUAVR) / 2015-2018 (CAVR)	54	54	Perceval S (21)/ Edwards Intuity (33)	Bioprosthetic valve (Hancock, Magna, Perimount)	Y	
17	Hartrumpf M [17]	2020	Germany	2012-2017	79	79	Perceval S / Edwards Intuity	Bioprosthetic valve (Mitroflow, Crown PRT, Perimount, Trifecta)	Y	
18	Herry M [18]	2020	France	2015-2018	256	668	Edwards Intuity	Bioprosthetic valve (Avalus, Crown, Magna, Mitroflow, Perimount, Trifecta)	Y	
19	Choi JW [19]	2021	Korea	2016-2018	641	2532	Perceval S (224) / Edwards Intuity (416)	Bioprosthetic valve	Y	
20	Erfe JM [20]	2021	The United States	2017-2018	4486	13215	Perceval S (1149) / Edwards Intuity (4727)	Bioprosthetic valve	Y	
21	Paparella D [21]	2021	Italy	2011-2019	430	860	Perceval S	Bioprosthetic valve (Hancock II, Mosaic, Carpentier-Edwards, Mitroflow, Crown)	Y	
22	Berretta P [22]	2022	18 large referral centers in Europe, Australia and Canada	2014-2022	2643	2643	Perceval S / Edwards Intuity	Bioprosthetic valve	Y	
23	Bottio T [23]	2022	Italy	2015-2020	144	141	Edwards Intuity	Bioprosthetic valve (Carpentier-Edwards Magna Ease)	Y	
24	Ono Y [24]	2022	Japan	2019-2021	65	65	Edwards Intuity	Bioprosthetic valve	Y	Y
25	Santarpino G [25]	2022	Italy	2010-2019	206	206	Perceval S	Bioprosthetic valve (Mosaic Ultra, Avalus)	Y	
26	D'Onofrio A [26]	2023 (ahead of print: 2022)	Italy	NA	252	123	Edwards Intuity	Bioprosthetic valve (Magna Ease)	Y	

Abbreviations: CAVR, conventional bioprosthetic aortic valve replacement; NA, not applicable; SUAVR, sutureless/rapid-deployment aortic valve replacement

Table S3 Quality assessment for randomized controlled trials included in the systematic review.

Study	Year of publication	Domain					Overall risk of bias
		1. Randomization process	2. Deviation from intended interventions	3. Missing outcome data	4. Measurement of outcome	5.Selection of the reported results	
Borger MA [1]	2015	High risk	Low risk	Low risk	Low risk	Low risk	High risk
Borger MA [2]	2016	High risk	Low risk	Low risk	Low risk	Low risk	High risk
Dedeilias P [3]	2016	Some concerns	Low risk	Low risk	Low risk	Low risk	Some concerns
Fischlein T [4]	2021	Some concerns	Low risk	Low risk	Low risk	Low risk	Some concerns

Table S4 Quality assessment for observational studies included in the systematic review.

Study	Year of publication	Domain							Overall risk of bias
		1. bias due to confounding	2. bias arising from measurement of the exposure	3. bias in selection of participants into the study	4. bias due to post-exposure interventions	5. bias due to missing data	6. bias arising from measurement of the outcome	7. bias in selection of the reported result	
Gilmanov D [5]	2014	Low risk	Low risk	Low risk	Low risk	Low risk	Low risk	Low risk	Low risk
Pollari F [6]	2014	Low risk	Low risk	Low risk	Low risk	Low risk	Low risk	Low risk	Low risk
Dalén M [7]	2015	Low risk	Low risk	Low risk	Low risk	Low risk	Low risk	Low risk	Low risk
Muneretto C [8]	2015	Low risk	Low risk	Low risk	Low risk	Low risk	Low risk	Low risk	Low risk
Forcillo J [9]	2016	Low risk	Low risk	Low risk	Low risk	Low risk	Low risk	Low risk	Low risk
Smith AL [10]	2017	Low risk	Low risk	Low risk	Low risk	Low risk	Low risk	Low risk	Low risk
Ensminger S [11]	2018	Low risk	Low risk	Low risk	Low risk	Low risk	Low risk	Low risk	Low risk
Nguyen A [12]	2018	Low risk	Low risk	Low risk	Low risk	Low risk	Low risk	Low risk	Low risk
Rahmanian PB [13]	2018	Low risk	Low risk	Low risk	Low risk	Low risk	Low risk	Low risk	Low risk
Repossini A [14]	2018	Low risk	Low risk	Low risk	Low risk	Low risk	Low risk	Low risk	Low risk
Rubino AS [15]	2018	Low risk	Low risk	Low risk	Low risk	Low risk	Low risk	Low risk	Low risk
Gotzmann M [16]	2020	Low risk	Low risk	Low risk	Low risk	High risk	Low risk	Low risk	High risk
Hartrumpf M [17]	2020	Low risk	Low risk	Low risk	Low risk	Low risk	Low risk	Low risk	Low risk
Herry M [18]	2020	Low risk	Low risk	Low risk	Low risk	Low risk	Low risk	Low risk	Low risk
Choi JW [19]	2021	Low risk	Low risk	Low risk	Low risk	Low risk	Low risk	Low risk	Low risk
Erfe JM [20]	2021	Low risk	Low risk	Low risk	Low risk	Low risk	Low risk	Low risk	Low risk
Paparella D [21]	2021	Low risk	Low risk	Low risk	Low risk	Low risk	Low risk	Low risk	Low risk
Berretta P [22]	2022	Low risk	Low risk	Low risk	Low risk	Low risk	Low risk	Low risk	Low risk
Bottio T [23]	2022	Low risk	Low risk	Low risk	Low risk	Low risk	Low risk	Low risk	Low risk
Ono Y [24]	2022	Low risk	Low risk	Low risk	Low risk	Low risk	Low risk	Low risk	Low risk
Santarpino G [25]	2022	Low risk	Low risk	Low risk	Low risk	Low risk	Low risk	Low risk	Low risk
D'Onofrio A [26]	2023 (ahead of print: 2022)	Low risk	Low risk	Low risk	Low risk	Low risk	Low risk	Low risk	Low risk

Table S5 Results of meta-analysis of 30-day outcomes: SUAVR vs. CAVR

	Overall AVR			Isolated AVR			Combined AVR		
**Outcomes**	**RR**	**95% CI**	**I^2 (%)**	**RR**	**95% CI**	**I^2 (%)**	**RR**	**95% CI**	**I^2 (%)**
Stroke	**1.21**	0.86-1.70	44	**1.81**	1.22-2.68	11	**0.87**	0.69-1.10	0
Atrial fibrillation	**0.88**	0.77-1.01	58	**0.85**	0.76-0.96	20	**0.86**	0.68-1.08	67
Major bleeding	**1.34**	1.11-1.62	0	**1.36**	1.12-1.64	0	**1.01**	0.43-2.41	0
Acute kidney injury	**1.07**	0.84-1.37	48	**0.82**	0.34-2.00	78	**1.27**	1.10-1.47	0
Pacemaker implantation	**2.52**	2.18-2.91	12	**2.86**	2.34-3.49	0	**2.34**	1.85-2.95	20
Paravalvular leakage	**1.25**	0.85-1.84	0	**1.31**	0.80-2.15	0	**1.18**	0.61-2.31	14
Death	**0.97**	0.84-1.12	0	**0.93**	0.68-1.27	0	**0.98**	0.83-1.16	0

Remarks:

1) The results of meta-analysis with random effect are reported as relative risk with 95% confidence interval.
2) The I^2 indicated the heterogeneity between the studies.

Abbreviations: CAVR, conventional bioprosthetic aortic valve replacement; CI, confidence interval; RR, relative risk; SUAVR, sutureless/rapid-deployment aortic valve replacement

References

1. Borger MA, Moustafine V, Conradi L, Knosalla C, Richter M, Merk DR, et al. A randomized multicenter trial of minimally invasive rapid deployment versus conventional full sternotomy aortic valve replacement. Ann Thorac Surg. 2015;99(1):17-25 doi: 10.1016/j.athoracsur.2014.09.022.
2. Borger MA, Dohmen PM, Knosalla C, Hammerschmidt R, Merk DR, Richter M, et al. Haemodynamic benefits of rapid deployment aortic valve replacement via a minimally invasive approach: 1-year results of a prospective multicentre randomized controlled trial. Eur J Cardiothorac Surg. 2016;50(4):713-20 doi: 10.1093/ejcts/ezw042.
3. Dedeilias P, Baikoussis NG, Prappa E, Asvestas D, Argiriou M, Charitos C. Aortic valve replacement in elderly with small aortic root and low body surface area; the Perceval S valve and its impact in effective orifice area. J Cardiothorac Surg. 2016;11(1):54 doi: 10.1186/s13019-016-0438-7.
4. Fischlein T, Folliguet T, Meuris B, Shrestha ML, Roselli EE, McGlothlin A, et al. Sutureless versus conventional bioprostheses for aortic valve replacement in severe symptomatic aortic valve stenosis. J Thorac Cardiovasc Surg. 2021;161(3):920-32 doi: 10.1016/j.jtcvs.2020.11.162.
5. Gilmanov D, Miceli A, Ferrarini M, Farneti P, Murzi M, Solinas M, et al. Aortic valve replacement through right anterior minithoracotomy: can sutureless technology improve clinical outcomes? Ann Thorac Surg. 2014;98(5):1585-92 doi: 10.1016/j.athoracsur.2014.05.092.
6. Pollari F, Santarpino G, Dell'Aquila AM, Gazdag L, Alnahas H, Vogt F, et al. Better short-term outcome by using sutureless valves: a propensity-matched score analysis. Ann Thorac Surg. 2014;98(2):611-6; discussion 6-7 doi: 10.1016/j.athoracsur.2014.04.072.
7. Dalén M, Biancari F, Rubino AS, Santarpino G, De Praetere H, Kasama K, et al. Ministernotomy versus full sternotomy aortic valve replacement with a sutureless bioprosthesis: a multicenter study. Ann Thorac Surg. 2015;99(2):524-30 doi: 10.1016/j.athoracsur.2014.08.028.
8. Muneretto C, Alfieri O, Cesana BM, Bisleri G, De Bonis M, Di Bartolomeo R, et al. A comparison of conventional surgery, transcatheter aortic valve replacement, and sutureless valves in "real-world" patients with aortic stenosis and intermediate- to high-risk profile. J Thorac Cardiovasc Surg. 2015;150(6):1570-7; discussion 7-9 doi: 10.1016/j.jtcvs.2015.08.052.
9. Forcillo J, Bouchard D, Nguyen A, Perrault L, Cartier R, Pellerin M, et al. Perioperative outcomes with sutureless versus stented biological aortic valves in elderly persons. J Thorac Cardiovasc Surg. 2016;151(6):1629-36 doi: 10.1016/j.jtcvs.2015.12.056.
10. Smith AL, Shi WY, Rosalion A, Yii M, O'Keefe M, Newcomb AE, et al. Rapid-Deployment Versus Conventional Bio-Prosthetic Aortic Valve Replacement. Heart Lung Circ. 2017;26(2):187-93 doi: 10.1016/j.hlc.2016.06.1202.
11. Ensminger S, Fujita B, Bauer T, Möllmann H, Beckmann A, Bekeredjian R, et al. Rapid Deployment Versus Conventional Bioprosthetic Valve Replacement for Aortic Stenosis. J Am Coll Cardiol. 2018;71(13):1417-28 doi: 10.1016/j.jacc.2018.01.065.
12. Nguyen A, Stevens LM, Bouchard D, Demers P, Perrault LP, Carrier M. Early Outcomes with Rapid-deployment vs Stented Biological Valves: A Propensity-match Analysis. Semin Thorac Cardiovasc Surg. 2018;30(1):16-23 doi: 10.1053/j.semtcvs.2017.09.002.
13. Rahmanian PB, Kaya S, Eghbalzadeh K, Menghesha H, Madershahian N, Wahlers T. Rapid Deployment Aortic Valve Replacement: Excellent Results and Increased Effective Orifice Areas. Ann Thorac Surg. 2018;105(1):24-30 doi: 10.1016/j.athoracsur.2017.07.047.
14. Repossini A, Fischlein T, Solinas M, Di Bacco L, Passaretti B, Grubitzsch H, et al. Stentless sutureless and transcatheter valves: a comparison of the hemodynamic performance of different prostheses concept. Minerva Cardioangiologica. 2018;66(2):180-90 doi: 10.23736/s0026-4725.17.04564-9.
15. Rubino AS, Santarpino G, De Praetere H, Kasama K, Dalén M, Sartipy U, et al. Early and intermediate outcome after aortic valve replacement with a sutureless bioprosthesis: Results of a multicenter study. J Thorac Cardiovasc Surg. 2014;148(3):865-71; discussion 71 doi: 10.1016/j.jtcvs.2014.03.052.
16. Gotzmann M, Wilbring M, Charitos E, Treede H, Silaschi M. Hemodynamic Comparison of Sutureless and Rapid-Deployment Valves with Conventional Bioprostheses. Thorac Cardiovasc Surg. 2020;68(7):584-94 doi: 10.1055/s-0039-1683426.
17. Hartrumpf M, Kuehnel RU, Schroeter F, Haase R, Laux ML, Ostovar R, et al. Clinical Short-Term Outcome and Hemodynamic Comparison of Six Contemporary Bovine Aortic Valve Prostheses. Thorac Cardiovasc Surg. 2020;68(7):557-66 doi: 10.1055/s-0038-1676853.
18. Herry M, Laghlam D, Touboul O, Nguyen LS, Estagnasié P, Brusset A, et al. Pacemaker implantation after aortic valve replacement: rapid-deployment Intuity® compared to conventional bioprostheses. Eur J Cardiothorac Surg. 2020;58(2):335-42 doi: 10.1093/ejcts/ezaa068.

19. Choi JW, Kim HJ, Kim JB, Lee S, Lim C, Chang BC, et al. Early and Two-year Outcomes after Sutureless and Conventional Aortic Valve Replacement: a Nationwide Population-based Study. J Korean Med Sci. 2021;36(9):e57 doi: 10.3346/jkms.2021.36.e57.
20. Erfe JM, Malaisrie SC, Andrei AC, Pham DT, Churyla A, Kruse J, et al. Outcomes of Sutureless/Rapid Deployment Valves Compared to Traditional Bioprosthetic Aortic Valves. Ann Thorac Surg. 2021;111(6):1884-91 doi: 10.1016/j.athoracsur.2020.07.034.
21. Paparella D, Santarpino G, Moscarelli M, Guida P, De Santis A, Fattouch K, et al. Minimally invasive aortic valve replacement: short-term efficacy of sutureless compared with stented bioprostheses. Interact Cardiovasc Thorac Surg. 2021;33(2):188-94 doi: 10.1093/icvts/ivab070.
22. Berretta P, Andreas M, Meuris B, Langenaeken T, Solinas M, Concistrè G, et al. Sutureless and rapid deployment versus sutured aortic valve replacement: a propensity-matched comparison from the Sutureless and Rapid Deployment International Registry. Eur J Cardiothorac Surg. 2022;62(2) doi: 10.1093/ejcts/ezac378.
23. Bottio T, Piperata A, Guariento A, Lorenzoni G, Cavicchiolo AG, Gemelli M, et al. Standard versus rapid-deployment aortic valve replacement and concomitant myocardial revascularization: 5-year bi-centre clinical outcomes. Eur J Cardiothorac Surg. 2022;62(5) doi: 10.1093/ejcts/ezac476.
24. Ono Y, Yajima S, Kainuma S, Kawamoto N, Tadokoro N, Kakuta T, et al. Early Outcomes of Intuity Rapid Deployment Aortic Valve Replacement Compared With Conventional Biological Valves in Japanese Patients. Circ J. 2022;86(11):1710-8 doi: 10.1253/circj.CJ-21-0959.
25. Santarpino G, Lorusso R, Peivandi AD, Atzeni F, Avolio M, Dell'Aquila AM, et al. In-Hospital Mortality and Risk Prediction in Minimally Invasive Sutureless versus Conventional Aortic Valve Replacement. Journal of Clinical Medicine. 2022;11(24) doi: 10.3390/jcm11247273.
26. D'Onofrio A, Cibin G, Lorenzoni G, Tessari C, Bifulco O, Lombardi V, et al. Propensity-Weighted Comparison of Conventional Stented and Rapid-Deployment Aortic Bioprostheses. Curr Probl Cardiol. 2023;48(1):101426 doi: 10.1016/j.cpcardiol.2022.101426.
